# Resection of a large multisegmental filum terminale ependymoma through a multisegmental hemilaminectomy

**DOI:** 10.3171/2023.6.FOCVID2349

**Published:** 2023-10-01

**Authors:** Paawan Bahadur Bhandari

**Affiliations:** Department of Surgery, Shree Birendra Hospital, Kathmandu, Nepal

**Keywords:** hemilaminectomy, myxopapillary ependymoma, intradural extramedullary tumor, filum terminale

## Abstract

This video demonstrates a gross-total resection of a multisegmental intradural extramedullary tumor using only multisegmental hemilaminectomy. The patient is a 21-year-old woman presenting with only backache. MRI of the lumbar spine demonstrates a large multisegmental heterogeneously enhancing intradural extramedullary tumor extending down from the eleventh dorsal vertebrae down to the fifth lumbar vertebrae. The surgical video demonstrates the technique of multisegmental hemilaminectomy and microsurgical resection of the tumor without posterior spinal instrumentation. Postoperatively, the patient had no neurological deficit and was discharged on postoperative day 5. Three-month postoperative MRI shows no residual disease or spinal deformity.

## Transcript

### 0:21

Here we present a case of a 21-year-old female who presented with backache and night pain and on neurological examination, she had no focal neurological deficit.

### 0:31

Contrast-enhanced MRI of the lumbar spine revealed a large heterogeneously enhancing lesion extending down from the eleventh dorsal vertebrae down to the fifth lumbar vertebrae. On axial images, the lesion was found occupying the entire spinal canal displacing the conus medullaris and the caudal equina toward the right side as shown by the yellow arrow in the top right image; the lesion is indicated by the red arrow.

### 0:54

With the patient in the prone position, a wide subperiosteal dissection of the paravertebral muscles was performed on the left side to expose the lamina of the tenth dorsal down to the fifth lumbar vertebra. A left-sided hemilaminectomy was performed by first drilling off the outer cortex of the lamina using a Rosen (cutting) burr. The inner cortex of the lamina was then drilled off using a diamond burr to thin out the bone in an eggshell technique. The thinned-out bones and the ligamentum flavum were carefully removed using Kerrison rongeur to expose the dura. The lateral edges were drilled and the spinous process was undermined using drill and Kerrison rongeur to expand the operative corridor. The epidural space was lined with oxidized cellulose and cotton patties.

### 2:05

The dura was opened in the middle of the hemilaminectomy using a sharp and blunt technique and dural retention sutures were applied. The arachnoid membrane over the cranial pole of the tumor was opened to release cerebrospinal fluid and decrease tension on the neural tissue. The arachnoid over the tumor was dissected off. Nerve roots were identified and carefully dissected off of the tumor.

### 2:30

The caudal pole of the tumor was identified and carefully elevated and brought into the operative field. Tumor samples were taken for biopsy. The tumor was soft, suckable, and was gradually debulked. The tumor capsule was gradually rolled into the field and carefully separated from the nerve roots. The dissected nerve roots was covered with cotton patties to prevent inadvertent injury.

### 3:16

The cranial end of the tumor was then debulked and the tumor was carefully dissected off of the spinal cord and the cauda equina using cotton patties to protect the nerve roots and apply counter traction.

### 3:38

The site of origin of the tumor from the dilated filum terminale is seen. The normal filum terminale is identified by the serpentine blood vessels overlying it. This is the zoomed-out view showing the operative field with the filum terminale seen overlying the cauda equina.

### 3:58

The cranial end of the diseased segment of the filum terminale was coagulated at low setting, cut, and then ligated. The diseased filum was carefully dissected and elevated away from the nerve roots. A dilated vein was found densely adherent to a nerve root which was sharply dissected off. The distal end of the filum terminale was then coagulated, ligated, and cut.

### 4:39

Here a nerve root exiting the spinal canal from the right side is demonstrated from the left-sided hemilaminectomy.

### 4:46

Here, a zoomed-out overview of the operative field is shown after a gross-total resection with normal neural tissues in situ. A water-tight dural closure was done using a 5-0 polyglactin suture without the use of dural sealant. The epidural space was lined with oxidized cellulose. Muscle and skin were closed in layers.

### 5:11

The patient developed transient urinary retention that resolved spontaneously on the 3rd postoperative day. She has no focal motor or sensory deficit and was ambulatory without support on the 1st postoperative day. She was discharged home on the 5th postoperative day. Histopathologically the tumor was a myxopapillary ependymoma, WHO grade 2.

### 5:23

This is the 3 months’ postoperative MRI showing no residual disease. Postoperative changes in the muscles is seen only on the left side; shown in the red circle in the upper right image. The normal muscles on the right side is shown in the green circle, and the preserved spinous process is indicated by the yellow arrow.

### 5:54

This video demonstrates the feasibility of gross-total resection of a multisegmental intradural extramedullary tumor using only multisegmental hemilaminectomy instead of a laminectomy or a laminoplasty. We believe that preservation of the posterior ligamentous complex and "minimally invasive" nature of this approach helps to maintain long-term stability of the spine without the use of posterior instrumentation especially in long-segment surgery. Thank you.

### 6:25

References^1–8^

## Disclosures

The author reports no conflict of interest concerning the materials or methods used in this study or the findings specified in this publication.

## Supplemental Information

### Patient Informed Consent

The necessary patient informed consent was obtained in this study.
